# The frequency and risk factors of deep vein thrombosis among surgical patients receiving thromboprophylaxis at Oshakati Intermediate Hospital: a six-year retrospective cohort study

**DOI:** 10.11604/pamj.2026.54.38.44882

**Published:** 2026-06-09

**Authors:** Tulipo Kisha Ipinge, Albertina Mariina Ndinelao Shatri

**Affiliations:** 1 School of Medicine, Hage Geingob Campus, University of Namibia, Windhoek, Namibia,; 2 Department of Human, Biology and Translational Medical Sciences, School of Medicine, University of Namibia, Windhoek, Namibia

**Keywords:** Venous thromboembolism, deep vein thrombosis, prophylaxis, human immunodeficiency virus, Caprini risk assessment tool, Namibia

## Abstract

**Introduction:**

venous thromboembolism (VTE) is the formation of clots in the deep veins. This is due to three main risk factors, namely: endothelial damage, hypercoagulability, and venous stasis. These may lead to complications affecting patient morbidity and mortality, as well as causing high healthcare costs. Prophylaxis and treatment are imperative to prevent recurrence and complications. There is, however, limited data, especially in the African context, on the efficacy of deep vein thrombosis (DVT) prophylaxis and its outcomes.

**Methods:**

ethical approval from the Ministry of Health and Social Services and the University of Namibia was obtained, and a retrospective review of 42 inpatient records that met the inclusion criteria from the targeted surgical departments from January 2017 to December 2022 was conducted.

**Results:**

despite prophylaxis, 44% of patients developed VTE, with the majority being from orthopedic surgery (50%). Most patients requiring prophylaxis were from orthopedic surgery, and among all patients requiring prophylaxis, 77% didn't receive any. The most common risk factors and complications were previous malignancy (28.6%) and pulmonary embolism (50%), respectively, and HIV infection correlated with VTE development (p=0.000147). The treatment for DVT followed the Namibian Standard Treatment Guidelines.

**Conclusion:**

these findings highlight the need to improve prophylactic treatment and the use of VTE prophylaxis tools, which are known to reduce VTE-related morbidity and mortality. Future research could explore the impact of inadequate VTE prophylaxis and the relationship between common comorbidities in sub-Saharan Africa and VTE prophylaxis.

## Introduction

Venous thromboembolism (VTE) is characterized by the formation of a clot or thrombus in a deep vein of the body, which may break off and move throughout the body [1]. Two main conditions fall into this spectrum: deep vein thrombosis (DVT) and pulmonary embolism (PE), together accounting for about 60000 - 100000 deaths each year globally [2]. A DVT happens when a blood clot appears in the deep veins of the limbs of the body, especially the lower limbs; however, these clots can also occur in the upper arm or pelvis, with some of the main symptoms being pain, swelling, redness, tenderness, warmth, and warmth of the affected limb [3,4]. Virchow's triad describes three primary risk factors for developing a DVT: endothelial damage, hypercoagulability, and venous stasis [5]. Many factors contribute to each of these categories, with surgery contributing to venous stasis [6]. According to the Centers for Disease Control and Prevention (CDC), in 2021, 50% of DVTs occurred due to hospitalization and surgery; therefore, any hospitalized patient, including those who have undergone surgery, is particularly at risk of developing DVTs [7].

Deep vein thrombosis can lead to devastating complications, which can be debilitating and costly for patients and the healthcare system as well [8]. Pulmonary embolism (PE) is a life-threatening condition that occurs when clots break off and migrate into the lungs, causing damage to pulmonary blood vessels and occluding blood flow through the lungs [5]. PEs may present with a sudden onset of chest pain, difficulty in breathing, coughing, palpitations, and anxiety [9]. Another complication is post-thrombotic syndrome (PTS), noted in 20-50% of DVT patients, characterized by frequent lower extremity edema, blood accumulation, changes in skin color, and ulcers of the affected area [10].

Prophylaxis is used to prevent the occurrence, recurrence, and consequences of DVT, done with the use of antithrombotic therapy consisting of Heparin and Warfarin, as well as non-pharmacological methods [2]. With adequate VTE prevention, healthcare systems can save costs and reduce morbidity and mortality among their patients. Although it was found that the frequency of DVT in Africa was highest among postoperative and postpartum patients [11], all the available data on VTE prevention, however, is restricted to countries in Europe and the United States of America. There is limited to no information regarding the frequency of DVT, the use of prophylaxis, and DVT complications associated with the treatment in Asia, South America, Africa, or most countries in sub-Saharan Africa [1].

Due to the lack of data, this study, the first of its kind in Namibia, aimed to fill this gap in the existing literature by determining the frequency of deep vein thrombosis among surgical patients receiving deep vein thrombosis prophylaxis at the Oshakati Intermediate Hospital, Oshakati, Namibia. This was achieved by: i) determining the standard treatment regimens used for DVT prophylaxis in each surgical department; ii) comparing the VTE prophylaxis treatment practices of each of the surgical departments with the recommended treatment in the Namibian Standard Guidelines [12] as well as the ninth American College of Chest Physicians´ (ACCP) Guidelines on VTE prevention [13]; iii) determining how many surgical patients in each department are given prophylactic treatment yearly (2017 - 2022); iv) determining the most common risk factor for DVT occurrence; v) determining how many postoperative patients in each department, despite receiving DVT prophylaxis develop DVTs; vi) establishing which surgical department accounts for the highest frequency of DVT development despite prophylaxis and; vii) determining the distribution of post-prophylactic DVT development in terms of sex and age.

In addition, this study establishes a foundation for future research about DVT, such as evaluating the effectiveness of current prophylactic or treatment regimens, therefore providing information on whether (and where) improvements can be made. Lastly, the results of this research can assist in identifying any major additional factors that contribute to the frequency of DVTs in patients postoperatively, which may aid in decision-making concerning the treatment of these patients in Namibia, especially.

## Methods

**Research design and setting:** a retrospective cohort study was conducted from January 2017 to December 2022 across four surgical departments at Oshakati Intermediate Hospital, Oshakati, Namibia. Approval from the University of Namibia and the Ministry of Health and Social Services was obtained (Ref no: TKI 2022). Approval was also obtained from the office of the superintendent at the intermediate hospital in Oshakati to conduct research at the hospital and to get patient files. The confidentiality of patients was maintained by excluding all personal information, such as identifiers or identification documents, from the research. Moreover, the collection of data and completion of the forms were done at the location where these files are stored to avoid carrying files around and potentially leaking each patient's personal information. The research also does not intend to cause any harm to any participant involved. All participants in the study were treated equally and fairly, and each member of the study population had a fair chance of being selected to participate.

**Study population and sampling:** the target population for this study comprised all medical records of surgical patients aged 18 years or older who had received prophylactic VTE treatment at Oshakati Intermediate Hospital. The sampled files were those available and that met the inclusion criteria. The following formula was used to estimate the sample size [14]. The following formula was used to estimate the sample size [14].

**Sample size calculation:** the sample size for this study was estimated using the formula for a single proportion:


$$n=\frac{Z^{2}p(1-p)}{e^{2}}$$

Where: n = desired sample size; Z = standard normal deviate corresponding to a 95% confidence interval = 1.96; p= estimated proportion of the population with deep vein thrombosis (DVT) = 0.051 (5.1%) [CDC, 2021]; 1 - p = proportion without DVT = 0.949; e = margin of error = 0.05 (5%); n=[(1.96)^2^*0.051*0.949]/(0.05)^2^=74.

Step-by-step: 1.96^2^= 3.8416; 0.051 x 0.949 = 0.0484; multiply: 3.8416 x 0.0484 =0.1857; divide by 0.05^2^=0.0025: 0.1857/0.0025 = 74.3. Thus, the estimated desired sample size was approximately 74 patients. Due to poor record-keeping and storage issues, only 42 patient files could be retrieved, which is below the calculated sample size and may reduce the precision of the study findings.

**The inclusion criteria included:** the surgical departments of Obstetrics and Gynecology, General Surgery, Orthopedic Surgery, and Neurosurgery; patients who have undergone surgery in Oshakati Intermediate Hospital from January 2017 to December 2022; all patients over the age of 18 years; all races and all sexes; patients who have received VTE prophylactic treatment either before or after surgery; patients whose records indicate the outcome of the treatment.

**The exclusion criteria included:** patients are taking antithrombotic treatment due to a past disease; patients who failed to complete their treatment or were lost to follow-up.

**Data collection tools and procedure:** the main ways in which data were collected were retrospective record reviews (hospital files), Google Forms to collect the required data, and additional tools: the Caprini risk assessment score, the 9^th^ ACCP guideline [13], and the Namibia Standard Treatment Guideline [12]. From August 2024 to October 2024, each surgical department was approached to obtain inpatient records for the years 2017 to 2022. The records were used to obtain patient file numbers, which were then used to retrieve the files from storage. The data collection process was conducted at the location where the patient´s files were stored from the 11^th^ to the 22^nd^ of December 2023. Each file was thoroughly reviewed to determine whether it met the inclusion and exclusion criteria. Of the files that satisfied the requirements, a Google Form (questionnaire) was completed, and relevant information, including age, sex, BMI, risk factors for VTE, comorbidities, prophylactic treatment, development of DVT post-surgery, and complications of VTE or treatment thereof, was noted for the study.

**Data analysis:** the data extracted from the patient´s files were automatically analyzed by Google Forms software and displayed as graphs and related percentages. The data were described using descriptive statistics and analyzed using Microsoft Excel and SPSS. The categorical data were expressed as frequencies and percentages to show the occurrence of DVTs post-treatment in each department, and a chi-square test was used to determine the relationship between the development of DVT post-prophylaxis and age, sex, and HIV status, with a significance level of α = 0.05.

## Results

**Overall results based on retrospective analysis:** a total of 328 file numbers were obtained from the respective departments for possible inclusion in the study; however, only 132 were received, whereas 196 were missing. Of the 132 files procured, 90 were excluded based on the exclusion criteria, leaving 42 that satisfied the inclusion criteria and were ultimately reviewed for the study (Figure 1). The 42 sampled files account for only 56.8% of the calculated desired sample size, possibly due to poor record-keeping and storage at the hospital. The sample distribution across the different departments was 21 (50%) for Obstetrician-Gynecologist (OBGYN), 13 (31%) for Orthopedic Surgery, 5 (12%) for Neurosurgery, and 3 (7%) for General Surgery.

**Figure 1:**
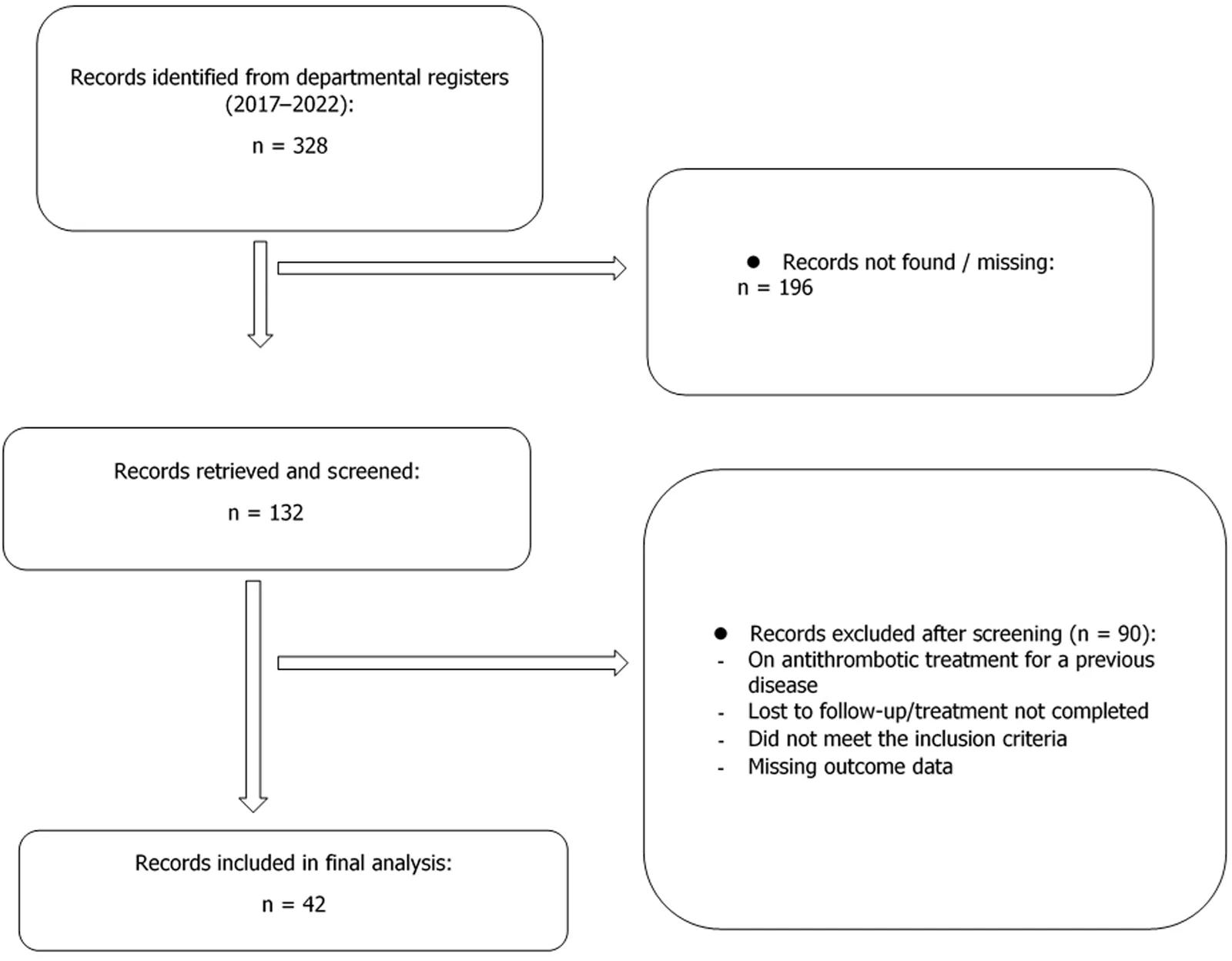
PRISMA-style flow diagram of surgical patient record selection at Oshakati Intermediate Hospital (2017-2022)

**Basic clinical characteristics of the cohort:** most files were for female patients, with a sex distribution of 16 males (38%) and 26 females (62%). The age categories were 18-40 years = 21, 41-60 years = 15, and 61+ years = 6, indicating that most of the study cohort was between 18 and 40 years old. None of the patients were reported to be smokers. Those who were HIV positive, a reported risk factor for DVT [15], were 22; HIV negatives were 15, and those with unknown HIV status were 5. It was noted that neither the weight nor the height of each patient was documented; therefore, BMI could not be calculated.

The common risk factors identified among the patients who would require prophylaxis for VTE (low, moderate, high risk) based on the Caprini risk assessment tool were: previous malignancy 28.6%, multiple trauma 16.7%, previous surgery 11.9%, history of unexplained/recurrent abortion 16.7%, pregnancy or postpartum 14.3%, bed rest/immobilization 11.8%. The frequency distribution of these variables is summarized in Table 1.

**Table 1:** basic clinical characteristics of the cohort

Variable	Categories	N (%)
Department	OBGYN	21 (50%)
	Orthopedic surgery	13 (31%)
	Neurosurgery	5 (12%)
	General surgery	3 (7%)
Age	18-40	21 (50%)
	41-60	15 (36%)
	61 and above	6 (14%)
Sex	Male	16 (38%)
	Female	26 (62%)
HIV status	Positive	22 (53%)
	Negative	15 (36%)
	Unknown	5 (11%)
Smoking status	Yes	0
	No	42 (100%)
	Unknown	0
BMI	Underweight	Unknown
	Normal weight	Unknown
	Overweight	Unknown
	Obese	Unknown
	Morbidly obese	Unknown
Risk factors	Previous/current malignancy	12 (28.6%)
	Multiple trauma 16.7%	7 (16.7%)
	History of unexplained or recurrent abortion	7 (16.7%)
	Pregnancy and postpartum	6 (14.3%)
	Previous surgery	5 (11.9%)
	Use of oral contraceptive pills	3 (7.1%)
	Bed rest/immobilization	2 (4.8%)

OBGYN: obstetrician-gynecologist; BMI: body mass index

**Risk stratification based on the Caprini risk assessment tool:** the distribution of samples based on risk categories for developing DVT post-prophylactic treatment, as categorized by the Caprini risk assessment tool, was set as: very low risk = 3, low risk = 10, moderate risk = 13, and high risk = 16, respectively. Based on the findings of this study, most patients had a high risk (39%) of developing VTE. This indicates that 93% of the cohort required some form of VTE prophylaxis. Table 2 outlines these results.

**Table 2:** risk stratification based on the Caprini risk assessment tool in each department

	OBGYN (N)	Orthopedic surgery (N)	Neurosurgery (N)	General surgery (N)	Total N (%)
Very low risk	1	2	0	1	3 (7%)
Low risk	7	1	2	0	10 (23%)
Moderate risk	12	0	1	0	13 (31%)
High risk	0	10	4	2	16 (39%)

OBGYN: obstetrician-gynecologist

**Prophylactic treatment:** in the study, 39/42 (93%) participants qualified for VTE prophylaxis; however, only 9/39 (23%) received it, and 77% of patients had not received it (Table 3). Of the nine who received prophylaxis, two were from OBGYN, five from orthopedic surgery, and one each from general surgery and neurosurgery, with all patients categorized as high risk according to the Caprini risk assessment tool and 9^th^ ACCP guideline. Moreover, 7/9 (78%) of those who received prophylaxis and DVT were HIV positive. Among the prophylactic treatments for VTE, Clexane was the only agent used for prophylaxis; there was no evidence that patients received mechanical prophylaxis for any patient, showing that only pharmacological methods of prophylaxis were employed, possibly exposing patients to a longer duration of treatment and higher doses, which may increase the risk of side effects associated with the use of Clexane.

**Table 3:** use of prophylactic treatment based on the ninth American College of Chest Physicians (ACCP) guideline

Risk categories	Qualified (%)	Received (%)	Did not receive (%)
Low risk	10 (23%)	0 (0%)	10 (33%)
Moderate risk	13 (31%)	0 (0%)	13 (44%)
High risk	16 (39%)	9 (100%)	7 (23%)
Total (%)	39 (93%)	9 (23%)	30 (77%)

**Clinical outcomes post prophylactic treatment:** of all patients who received DVT prophylaxis, 4 (44%) developed VTE post-prophylactic treatment, of which 2 (50%) were from orthopedic surgery, 1 (25%) from OBGYN, and 1 (25%) from general surgery. No neurosurgical patients developed DVT despite prophylaxis; Table 4 outlines these results. Based on statistical analysis, no significant relationship was found between age, sex, and the development of VTE (p-value of p=0.870 and p=0.562, respectively). There was, however, a significant relationship between VTE development and RVD reactivity (p=0.000147).

**Table 4:** distribution and clinical characteristics of patients who developed DVT post-prophylactic treatment

Variable	Variable subgrouping	OBGYN	Orthopedic Surgery	Neurosurgery	General Surgery	Totals (%)	P-value
Age	18-40	1	3	0	0	4 (45%)	0.870
	41-60	1	1	0	1	3 (33%)	
	61 and above	0	1	1	0	2 (22%)	
Sex	Male	0	5	0	1	6 (67%)	0.562
	Female	2	0	1	0	3 (33%)	
HIV status	Positive	2	4	0	1	7 (78%)	0.000147*
	Negative	0	1	0	0	1 (11%)	
	Unknown	0	0	1	0	1 (11%)	

* : significant correlation; OBGYN: obstetrician-gynecologist; DVT: deep vein thrombosis

**Complications of VTE/treatment:** in this study, a total of 4 patients developed complications after the development of DVT related either to DVT treatment or the DVT itself. The complications ranged from PE 2 (50%) to venous insufficiency 1 (25%) and warfarin toxicity 1 (25%).

## Discussion

This study assessed the frequency of postoperative deep vein thrombosis (DVT) and associated risk factors among surgical patients receiving thromboprophylaxis at Oshakati Intermediate Hospital. To our knowledge, this is the first study in Namibia to evaluate the use of venous thromboembolism (VTE) prophylaxis across multiple surgical departments, providing locally relevant data to inform clinical practice and guideline development.

The findings indicate that most patients were at moderate to high risk of VTE according to the Caprini risk assessment tool, yet thromboprophylaxis use was suboptimal. Similar gaps in the implementation of VTE prophylaxis have been reported in other African settings. Studies from South Africa, Ethiopia, and Nigeria have documented low rates of appropriate prophylaxis among surgical patients despite a high prevalence of VTE risk factors, underscoring systemic challenges in risk assessment, guideline adherence, and resource availability across sub-Saharan Africa [16,17].

It was evident that prophylactic treatment was most commonly used in the Orthopedic Surgery Department and least commonly in Obstetrics and Gynecology. There has also been no evidence of the use of mechanical prophylactic agents, which have been shown to further reduce VTE risk when used in addition to pharmacological agents [16]. Although it seems that only patients who were high risk received prophylactic treatment, many who weren´t at high risk did not receive any prophylaxis; therefore, it is unclear establish whether or not risk stratification tools are used for patients at risk of DVT despite scientific evidence reinforcing that they are of advantage, and because the Namibia Standard Treatment Guideline doesn´t show the use of any risk stratification and treatment based on those risks, it is unclear as to why precisely some patients are placed on prophylactic treatment and some are not. It would, however, not be surprising that none are in use, as it has been pointed out previously that the use and implementation of these risk stratification tools are low globally [16].

Although 67% of patients who developed VTE post-prophylactic treatment were male, and 45% of these patients were between the age range of 18-40 years, there was no significant relationship found between age or sex and the development of VTE post-prophylactic treatment. However, age and sex are not risk factors for DVT. Differences in sex influence the first occurrence and site of VTE, such as men more commonly presenting with proximal DVT and women presenting with more distal DVT in the lower limbs [18].

A significant association was observed between HIV positivity and the development of VTE, aligning with studies from South Africa and other parts of sub-Saharan Africa that have identified HIV infection as an independent risk factor for thrombosis due to chronic inflammation, endothelial dysfunction, and antiretroviral therapy-related effects [15]. This underscores the importance of incorporating HIV status into VTE risk stratification in regions with high HIV prevalence.

Mechanical prophylaxis was not documented in any patient, with pharmacological prophylaxis limited to low-molecular-weight heparin. Evidence from African and international literature suggests that combined mechanical and pharmacological prophylaxis may provide superior protection against VTE, particularly in high-risk surgical patients [16]. The absence of mechanical prophylaxis in this setting may reflect resource constraints or limited awareness and warrants further investigation.

Overall, the findings highlight the need for improved implementation of standardized VTE risk assessment tools, better adherence to thromboprophylaxis guidelines, and strengthened medical record management practices. However, these results should be interpreted with caution due to several limitations, including a high number of missing patient records, which produced a small, potentially non-representative sample with limited statistical power. The retrospective design relied on the completeness and accuracy of existing records, resulting in missing data for key variables such as body mass index and mechanical prophylaxis use and increasing the risk of selection or misclassification bias. In addition, the small number of patients who received thromboprophylaxis limits reliable estimation of VTE rates and precludes causal inference. Future prospective studies with larger sample sizes and more complete data are therefore needed to better estimate VTE incidence and evaluate the effectiveness of prophylactic strategies in Namibian surgical populations.

## Conclusion

Venous thromboembolism remains a significant concern among surgical patients at Oshakati Intermediate Hospital, with most patients at moderate to high risk, yet many do not receive prophylaxis. HIV was associated with VTE despite prophylaxis, whereas age and sex were not. Common risk factors included previous malignancy, multiple traumas, and recurrent or unexplained abortion. Pulmonary embolism was the most frequent complication. These findings highlight the need for routine use of risk-stratification tools, adherence to prophylaxis guidelines, and improved medical record management to enable timely interventions. Further research with larger samples is needed to evaluate prophylaxis practices and the impact of comorbidities, such as HIV, in the Namibian surgical population.

### What is known about this topic

- The frequency of DVT in Africa was highest among postoperative and postpartum patients; all of the available data on VTE prevention, however, is restricted to countries in Europe and the United States of America; however, there is limited to no information regarding the frequency of DVT, the use of prophylaxis, and DVT complications associated with the treatment in Asia, South America, and a few African countries.

### What this study adds

- This is the first study conducted in Namibia to identify the frequency and risk factors of deep vein thrombosis in surgical patients who receive deep vein thrombosis prophylaxis, thereby filling the existing knowledge gap in Namibia;
- High prevalence of VTE risk factors in the cohort: the data show that 93% of patients required prophylaxis, with 39% categorized as high risk based on the Caprini score; common risk factors included prior malignancy (28.6%), multiple trauma (16.7%), pregnancy or postpartum status (14.3%), and HIV positivity (52% of all known statuses);
- Severe underuse of prophylaxis: although 39 out of 42 patients qualified for prophylaxis, only 9 (23%) received it, highlighting a clear gap in adherence to established guidelines in clinical practice; lack of multimodal prophylaxis: all prophylaxis administered was pharmacologic (Clexane only); no mechanical methods were used at all, indicating a substantial resource and practice deficiency within the institution; poor clinical outcomes despite prophylaxis: of the nine patients who received prophylaxis, four (44%) still developed VTE; complications such as pulmonary embolism, venous insufficiency, and warfarin toxicity further emphasize the clinical consequences of inadequate or inconsistent VTE prevention and monitoring.
